# Protean Drainage Patterns of the Left Renal Vein: A Cadaveric and Retrospective Clinical Study on the Surgical Implications and Technical Feasibility

**DOI:** 10.7759/cureus.63037

**Published:** 2024-06-24

**Authors:** Amit K Shreevastava, Rajat S Das, Amit Mishra

**Affiliations:** 1 Anatomy, All India Institute of Medical Sciences, Raebareli, Raebareli, IND; 2 Urology, All India Institute of Medical Sciences, Raebareli, Raebareli, IND

**Keywords:** renal transplant, donor nephrectomy, left common iliac vein, inferior vena cava, delayed confluence of veins, retroaortic, circumaortic, variable anatomical drainage pattern, left renal vein

## Abstract

Background: The diverse drainage patterns of the left renal vein (LRV), often with asymptomatic congenital anomalies, present considerable challenges in renal and retroperitoneal surgical contexts. The potential for significant bleeding and subsequent renal compromise upon vascular injury highlights the need for increased surgical awareness.

Objective: This study investigates the LRV's variable anatomical drainage patterns and morphometry. It also evaluates the embryological factors contributing to these variations and discusses their surgical implications and technical considerations.

Methods: Anatomical dissections were conducted on 21 adult human cadavers within the Department of Anatomy. Concurrently, a retrospective analysis was conducted on 15 patients who underwent various retroperitoneal surgical interventions in the Urology Department. Demographic variables and intraoperative findings were recorded and analyzed.

Results: Dissection analysis predominantly identified preaortic LRVs in 18 cadavers. Notable anatomical variations included a circumaortic left renal vein (CLRV), a delayed preaortic confluence of extrahilar duo LRVs, and an extrahilar tetramerous confluence with a retroiliac topography. The majority of LRVs usually end in the inferior vena cava. However, an extrahilar tetramerous variant had an unusual drainage pathway. Out of 15 cases, three (20%) had a retroaortic left renal vein (RLRV). One patient with a nonfunctioning kidney had type 1 RLRV, and another patient with pelvic ureteric junction obstruction had type 4 retroiliac left renal vein (RILRV). In both of these patients, symptoms were relieved after surgery. In a young patient with left varicocele and microscopic hematuria who had type 2 RLRV, symptoms resolved spontaneously after a few months.

Conclusion: A thorough understanding of the variable anatomical drainage patterns of the LRV is crucial for surgeons. Accurate preoperative identification can provide valuable insights, potentially leading to improved surgical outcomes in renal procedures.

## Introduction

The embryogenesis of the inferior vena cava (IVC) and the left renal vein (LRV) is a complex and dynamic process [1]. It originates from the intricate remodeling of the anastomotic channels among the paired subcardinal, supracardinal, and posterior cardinal veins [1,2]. Faulty remodeling within these channels can result in diverse morphologies and topographies of the LRV [1,2,3,4]. Variable drainage patterns of the LRV are typically asymptomatic congenital anomalies, often remaining undetected until discovered incidentally during preoperative imaging, surgical interventions, cadaveric dissections, or autopsies [3,4]. The LRV is longer than the right renal vein. It extends across the abdominal aorta to drain into the IVC. This extended pathway likely contributes to the variability in its drainage patterns. The preaortic LRV is the usual anatomical configuration. When the LRV courses behind the aorta, it is called a retroaortic left renal vein (RLRV). A circumaortic left renal vein (CLRV) encircles the aorta anteriorly and posteriorly. LRV variations occur in 0.8-10.2% of the population [5]. RLRV and CLRV incidence varies between less than 1% to 10% and below 1% to above 15%, respectively [6]. In cases of donor nephrectomy in renal transplant, “warm ischemia time” is a very critical factor that has a long-term prognostic impact on renal graft survival, and the presence of multiple vessels and small length of vessels may prolong the ischemia time due to technical difficulty in procurement and wastage of time in multiple vessel anastomosis during bench surgery [7]. The left side kidney, for its longer venous pedicle, is often preferred to the right kidney for the obvious convenience of anastomose, resulting in less ischemia time of renal graft. The topography of the LRV is also of great clinical importance - whether preaortic or retroaortic in planning donor nephrectomy [7,8].

Meager information is available in the literature regarding the impact of anomalous LRVs on the outcome of simple nephrectomy, pyeloplasty, laparoscopic left donor nephrectomy, and subsequent transplantation. Raman et al. have classified renal vein anomalies into major or minor subtypes. Major renal vein anomalies are those that result in altered surgical management, including the creation of additional venous anastomosis in the recipient. The major renal vein anomalies include supernumerary veins and the presence of a late venous confluence, circumaortic or retroaortic renal veins, and a left-sided IVC or a duplicated IVC [9]. Minor venous anomalies affect the planning of donor laparoscopic dissection but do not change the venous anastomosis procedure in the recipient. These anomalies include issues with the lumbar, gonadal, adrenal, and/or retroperitoneal veins, as well as the large gonadal and lumbar veins (>5 mm) and their connection with the main or branch renal veins [10]. The presence of circumaortic or retroaortic renal veins is technically challenging in donor nephrectomy and hence considered a relative contraindication by some due to a greater propensity for inadvertent renal vein injuries, and many series have reported renal graft loss due to iatrogenic vascular complications in these cases [11,12]. Thus, the unpredicted anatomical topography of the LRV makes it a matter of utmost concern from the point of the applied surgical aspect. This has an undeniable bearing on the surgical implication and technical feasibility.

Novelty

The presence of unusual branches of the LRV after embryological development is rare in itself. This study, which combines insights and expertise from two departments approaching the phenomenon from different perspectives and its implications for patients, is even rarer. To date, this innovative approach has not been explored in existing literature, and we aim to convey this through our study. This study will strengthen the knowledge gained from cadaveric dissection and surgical procedures, which is crucial for a comprehensive understanding of potential anatomical variations and to explore all technical possibilities for achieving desired surgical outcomes.

Objectives

Primary Objective

This study aimed to study the LRV's variable anatomical drainage pattern and morphometry and to evaluate the possible embryological factors responsible for the variable drainage pattern of the LRV along with its surgical implications and technical feasibilities.

Secondary Outcome

This study aimed to reinforce the anatomical knowledge for better surgical applications by urologists so that they may be vigilant before embarking upon retroperitoneal surgeries, donor nephrectomies, and renal transplants.

## Materials and methods

Ethical approval was taken from the Institutional Ethics Committee of All India Institute of Medical Sciences (AIIMS), Raebareli (approval no. 2024-10-IMP-EXP-7). For the present study, 21 adult human cadavers, which included 18 males (86%) and three females (14%), were dissected in the Department of Anatomy. Standard dissection protocol was followed during the entire procedure. Cadavers with deformed kidneys or with congenital anomalies, such as horseshoe kidney, congenital or acquired absence of one kidney, tumors of kidneys or injured renal vessels/IVC, or evidence of surgery, i.e., partial nephrectomy, were excluded from the study. A digital vernier caliper (Freemans FDC 150; FMI Ltd., Doraha, India), with an accuracy of 0.1 mm, calculated the morphometric details of the relevant anatomical structures. Anatomical details were carefully observed, recorded, and digitally photographed.

A total of 15 patients, five male (33%) and 10 female (66%) patients, who underwent various retroperitoneal surgeries from January 2023 to April 2024 were retrospectively reviewed. All patients with left kidney disease and symptoms were included in the study and thoroughly investigated with a renal scan and computed tomography (CT) scan of the abdomen and pelvis. CT angiography was done only in cases where anomalous renal vasculature was suspected. Informed consent was taken from all the patients who underwent surgery. Patients with right renal vein anomalies were excluded from the study. The data were analyzed in a retrospective and deidentified manner from patient case records. In cases of nonfunctioning kidney (NFK)/poorly functioning kidney (PFK), a standard retroperitoneoscopic approach was used with the patient in flank position and three ports - one camera port (12 mm) in the midaxillary line, one anterior axillary port (5 mm), and one renal angle port (10 mm). In all cases of PUJ obstruction, the transperitoneal approach was utilized with a patient in 70 degrees right lateral position along with the routine three-port method - one pararectal camera port (10 mm) and two assistant ports in the triangulation position (one 5 mm assistant port for the left hand and another 10 mm assistant port for suturing with the right hand).

## Results

Anatomical study results

The dissection analysis revealed the presence of preaortic LRVs in 18 cadavers. The preaortic LRVs had an average length of 7.6 cm ± 0.63 cm and a diameter of 1.5 cm ± 0.19 cm across a total of 18 cases. Some notable anatomical variations included a CLRV, a delayed confluence of double LRVs, and an extrahilar tetramerous confluence with a retroiliac topography.

**Figure 1:**
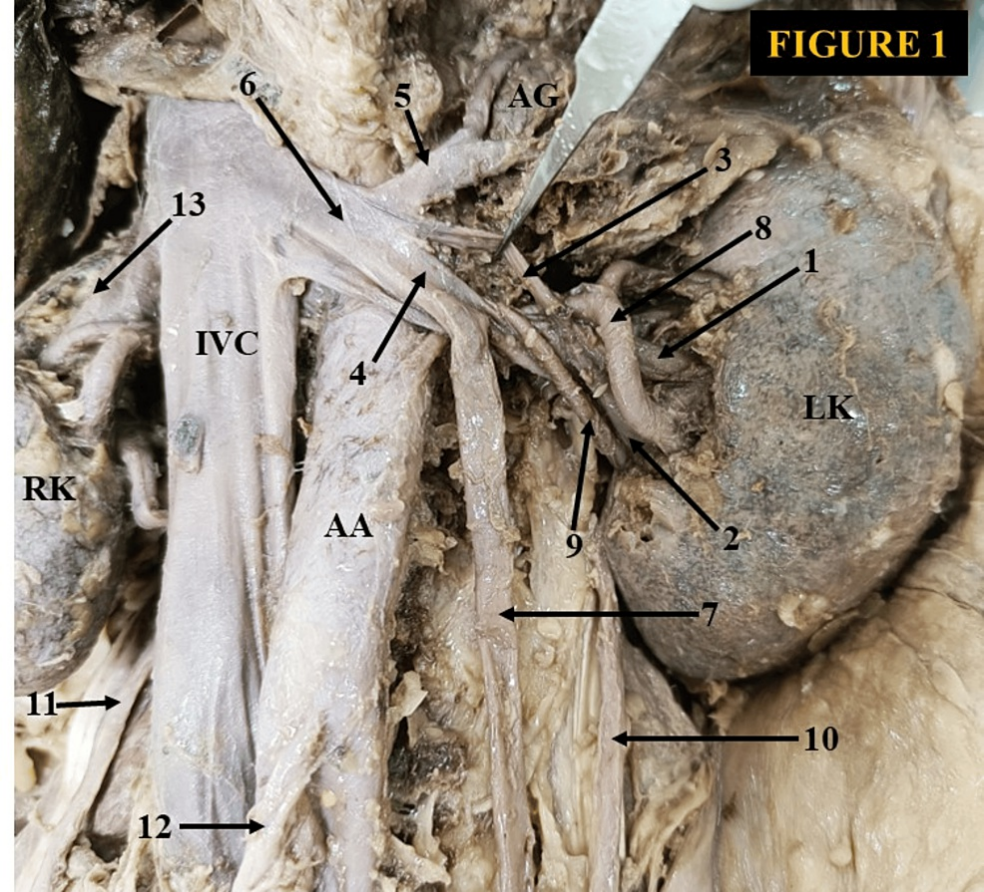
A delayed preaortic confluence of extrahilar double left renal veins (LRVs). 1) upper left hilar renal vein, 2) lower left hilar renal vein, 3) superior left extrahilar LRV, 4) inferior left extrahilar LRV, 5) left adrenal vein, 6) the main segment of a delayed confluence of LRV, 7) left ovarian vein, 8) main left renal artery, 9) accessory left renal artery, 10) left ureter, 11) right ureter, 12) inferior mesenteric artery, 13) right renal vein. LK: left kidney, RK: right kidney, IVC: inferior vena cava, AA: abdominal aorta, LRV: left renal vein, AG: adrenal gland, SMA: superior mesenteric artery

**Figure 2:**
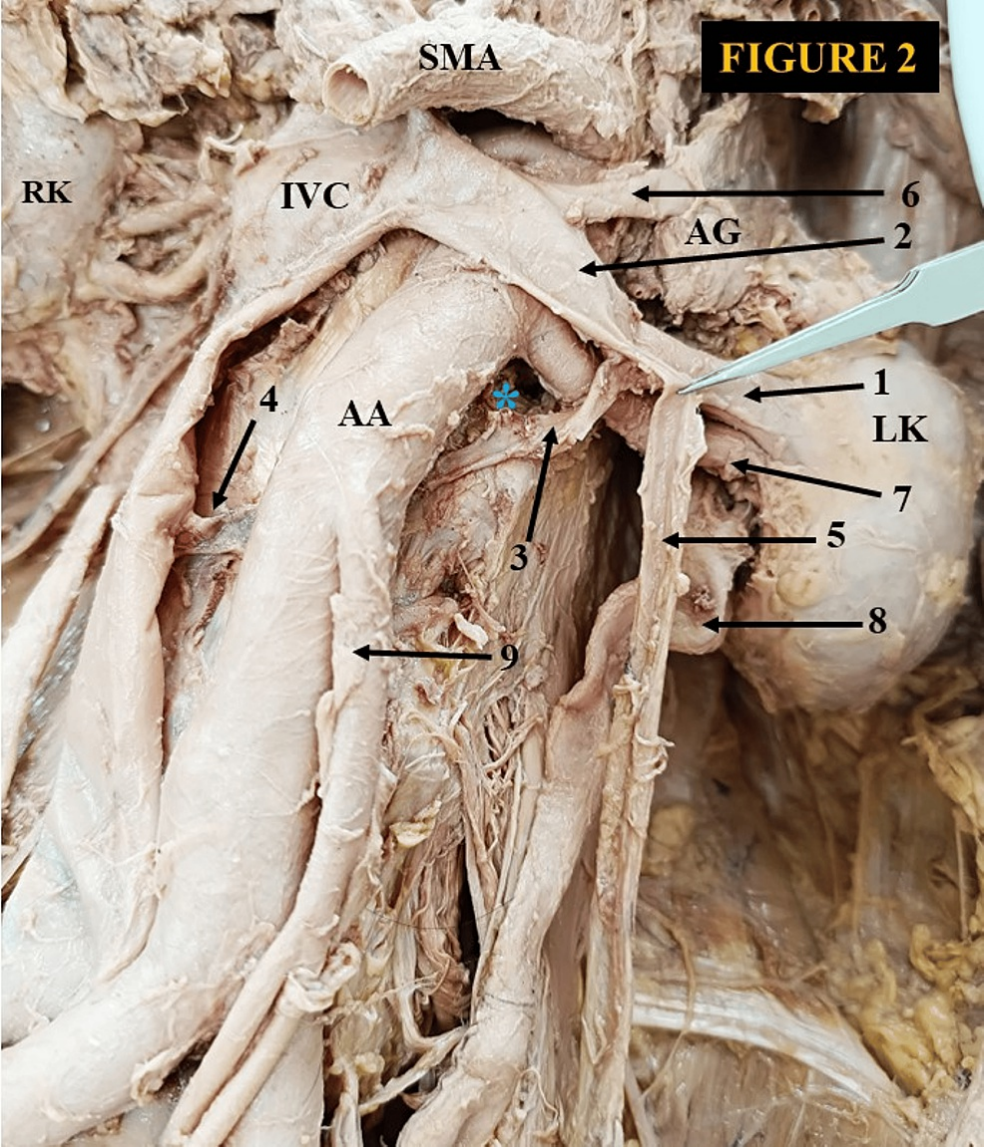
A circumaortic left renal vein (CLRV). 1) left hilar renal vein splitting to form a circumaortic left renal vein, 2) preaortic venous limb, 3) retroaortic venous limb, 4) retroaortic venous limb joining the IVC, 5) left testicular vein, 6) left adrenal vein, 7) left renal artery, 8) left ureter, 9) inferior mesenteric artery. LK: left kidney, RK: right kidney, AA: abdominal aorta, IVC: inferior vena cava, AG: adrenal gland, SMA: superior mesenteric artery. * Indicates the anastomosis of retroaortic venous limb and the hemiazygos vein.

In Figure 1, we observed two independent hilar LRVs that joined to form a common venous trunk, which then split into superior and inferior extrahilar LRVs. The duo extrahilar LRVs join with the left adrenal vein and organize a delayed confluence in front of the abdominal aorta, approximately 1.4 cm from the left lateral margin of the IVC [10]. The confluence of veins drains horizontally as a single venous channel into the IVC at the L1 vertebral level. The main venous channel was the inferior extrahilar LRV, which received the left ovarian vein. In this case, the main and accessory renal arteries are observed ventrally to the two LRVs. A CLRV was discovered in a male cadaver (Figure 2).

**Figure 3:**
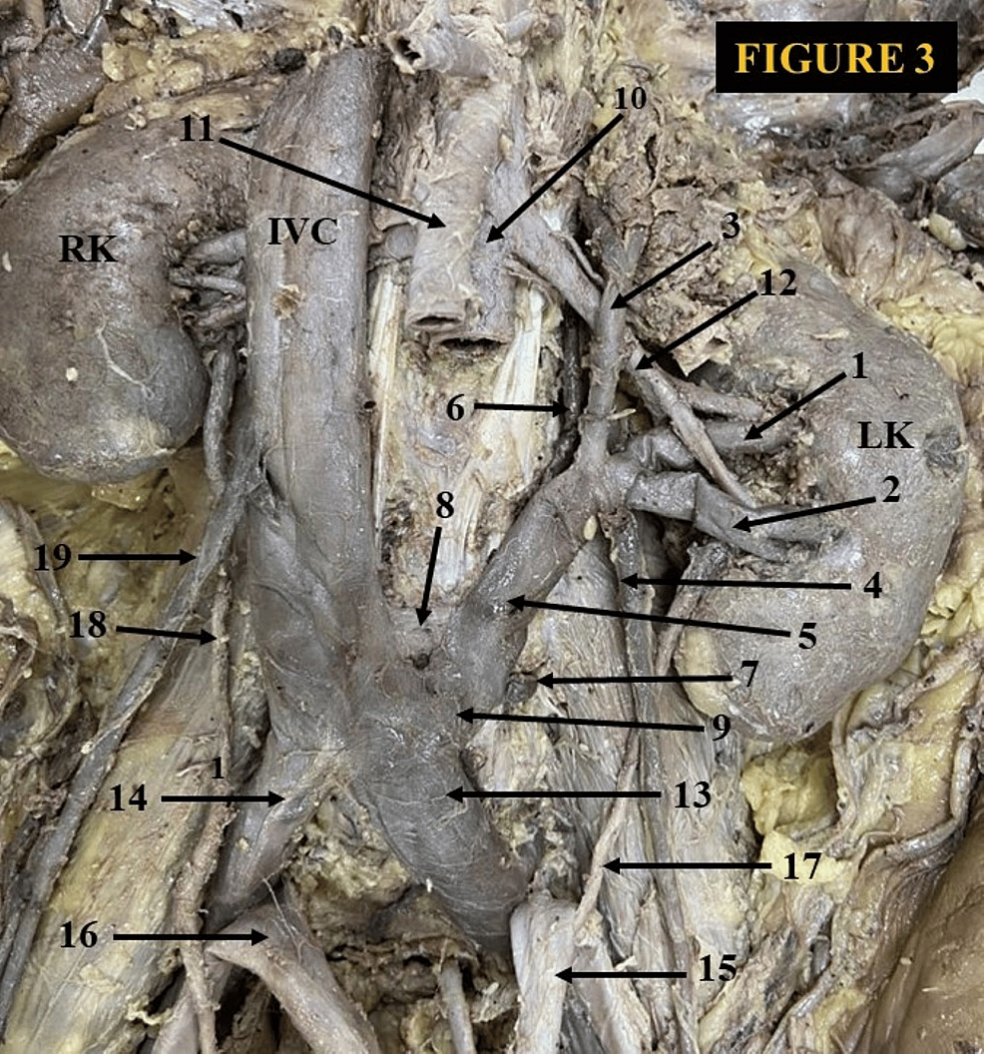
An extrahilar tetramerous retroiliac left renal vein (LRV). The left kidney is ectopic. 1) upper hilar left renal vein, 2) lower hilar LRV, 3) left adrenal vein, 4) left testicular vein, 5) retroiliac LRV, 6) hemiazygos vein, 7) 4th lumbar vein, 8) splitting segment of retroiliac LRV draining into the inferior vena cava, 9) splitting segment of the retroiliac LRV draining into the left common iliac vein, 10) abdominal aorta, 11) superior mesenteric artery, 12) left renal artery, 13) left common iliac vein, 14) right common iliac vein, 15) left common iliac artery, 16) right common iliac artery, 17) left ureter, 18) right ureter, 19) right testicular vein. LRV: left renal vein, IVC: inferior vena cava, LK: left kidney, RK: right kidney

The CLRV started as a single vein and then split into an anterior and a posterior venous limb. The anterior venous limb traveled slightly upward, crossed the abdominal aorta anteriorly, and drained into the IVC at the L1 vertebral level. The anterior (preaortic) venous arch receives the left adrenal and the testicular vein. The posterior venous limb took a downward oblique course, passed behind the abdominal aorta, and drained into the IVC at the L3-L4 vertebral level. The posterior limb of the CLRV was also connected to the hemiazygos vein. The anterior limb of the circumaortic vein measured 7.1 cm in length and 1.4 cm in width, while the posterior limb was 6.8 cm long and 0.7 cm wide. The anterior aortic segment was the main venous channel. The left kidney was positioned slightly below the right kidney in this case. We also observed an unusual configuration of the LRV in a male cadaver. The main segment of the LRV formed from the confluence of two renal veins, one adrenal vein, and a testicular vein outside the hilum (Figure 3).

In its trajectory, it connected with the hemiazygous vein at the proximal end and received the 4th lumbar vein at its distal point. Further down, it is divided into branching paths, converging with both the left common iliac vein and the lower segment of the IVC. Characterized by a downward oblique path, it navigated behind the left common iliac artery before culminating in its ultimate drainage points. We named this unique vein the extrahilar tetramerous retroiliac left renal vein (RILRV) (Figure 3). In this case, the left kidney was ectopic, with the upper pole at the level of L3 and the lower pole at the L5 vertebral level. The main segment of the tetramerous vein measured 6.7 cm in length with a diameter of 1.5 cm.

Surgical results

Out of 15 patients who were found eligible for the study, there were five male and 10 female patients. The mean age of the patients was 33 years (Table 1). Out of 15 patients, three patients (20%) had a positive finding in the form of RLRV.

**Table 1:** Demographic variables of surgical patients

Pt. no.	Age (years)	Sex	Presenting symptoms	Diagnosis	Operative time	Estimated blood loss	Anatomical anomalies
1	40	F	Flank pain,hematuria	PFK	3:30	150	RLRV type I
2	26	F	Recurrent flank pain, microscopic hematuria	PUJO	4:00	160	RLRV type IV
3	23	M	Lt Flank pain, Lt inguinal pain, microscopic hematuria	Lt varicocele	Conservative management	NA	RLRV type II

**Figure 4:**
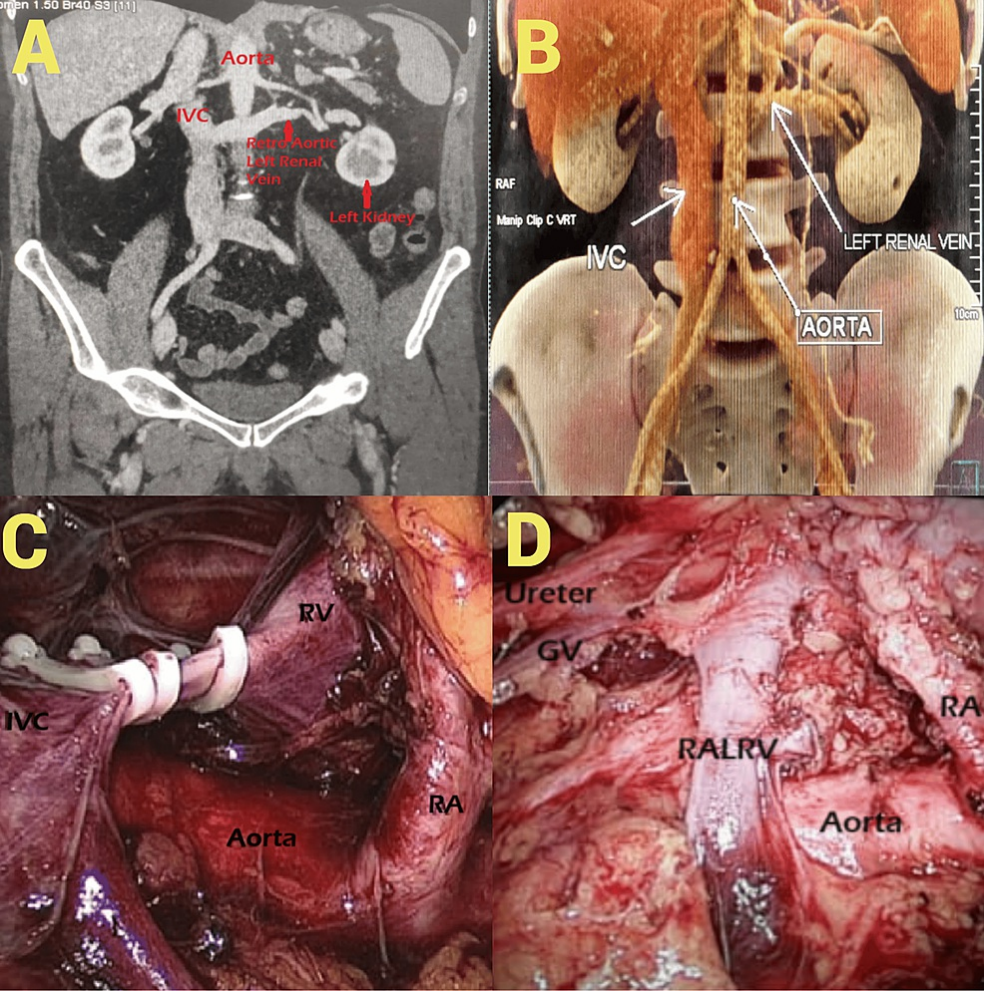
Lt PFK with RLRV type 1 A: CT angiogram showing the left renal vein draining into the IVC after passing posterior to the aorta. B: Reconstructed CT abdomen and pelvis image depicting the retroaortic course of the left renal vein. C: Lap retroperitoneoscopic image portraying the retroaortic course of the left renal vein (RALRV) draining into the IVC. D: Lap retroperitoneoscopic image with a dropped-down kidney laterally to display the retroaortic course of the left renal vein (RALRV) more vividly. PFK: poorly functioning kidney, RV: renal vein, RA: renal artery, IVC: inferior vena cava, RLRV: retroaortic left renal vein, GV: gonadal vein

The most common presenting feature was left flank pain along with recurrent UTI. Patients with concomitant renal and ureteric calculi also had on-and-off hematuria. One young patient presented with left flank pain on and off along with left inguinal pain and microscopic hematuria diagnosed as left varicocele grade II. Out of seven patients with left NFK/PFK who underwent left nephrectomy, one had type 1 RLRV (Figure 4A-4D).

**Figure 5:**
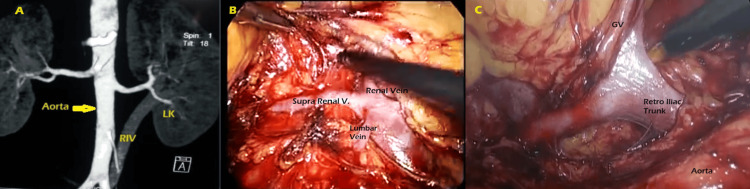
Lt RILRV type IV (retroiliac renal vein) with PUJO, intrarenal pelvis, and left PFK A: CT angiogram showing the oblique course of the left retroiliac renal vein (RIV) in a case of PUJ obstruction coursing posterior to the left common iliac artery. B: Laparoscopic transperitoneal image showing the course of three tributaries of the left renal vein (left suprarenal, lumbar vein, and parallel to the aorta trunk). C: Laparoscopic image showing the two tributaries of left renal vein emanating from straight parallel to the aorta trunk - gonadal vein and retroiliac trunk. PUJO: pelviureteral junction obstruction, PFK: poorly functioning kidney, GV: gonadal vein, LK: left kidney, RIV: retroiliac renal vein

He presented with left flank pain and microscopic hematuria; his symptoms subsided after the procedure. Seven patients had left pelvic ureteric junction obstruction, and one of these had a poorly functioning kidney(PFK), so dissection was done similar to nephrectomy. In this case, the cause of PUJ obstruction was left RILRV type IV (Figure 5A-5D).

**Figure 6:**
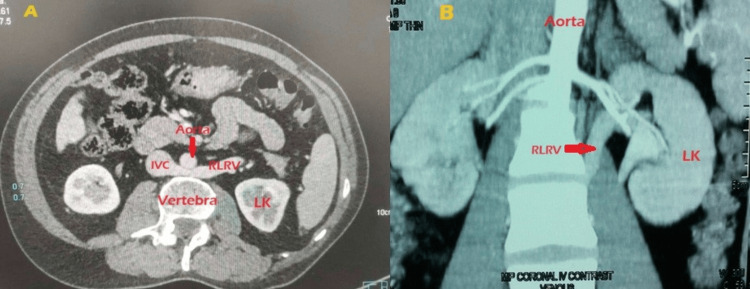
Lt RLRV type II Lt varicocele with flank pain and microscopic hematuria A: Axial CT abdomen image at the level of the left renal vein (RLRV) origin, showing its compression between the aorta and vertebra, leading to posterior nutcracker syndrome (NCS). B: Coronal CT abdomen and pelvis image depicting the oblique course of left renal vein (RLRV) posterior to aorta. RLRV: retroaortic left renal vein, IVC: inferior vena cava, LK: left kidney

The pelvis was intrarenal; it was dissected, dismembered, and transposed anterior to the vein while preserving the vein. Then, Anderson-Hynes pyeloplasty was performed as a significant proportion of parenchyma appeared normal intraoperatively. The patient was completely relieved after the pyeloplasty. The patient with left varicocele and poorly functioning kidney on evaluation had type II RLRV on CT angiography; he was managed conservatively, and his symptoms resolved after a few months spontaneously (Figure 6A, 6B).

The mean operative duration was 190 minutes in the nephrectomy group and 250 minutes in patients who underwent pyeloplasty. The mean blood loss was 160 ml in the nephrectomy group and 130 ml in the pyeloplasty group. There were no major complications encountered during the procedure.

## Discussion

**Figure 7:**
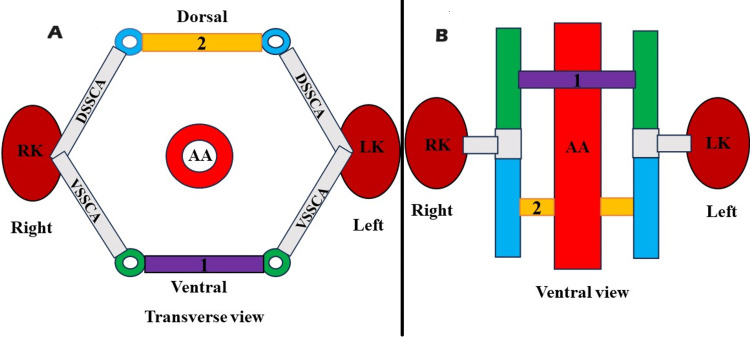
A: Schematic representation of a circumaortic venous collar (renal collar) during the early embryonic period (transverse view). B: Schematic representation of a circumaortic venous collar (renal collar) during the early embryonic period (ventral view). 1) intersubcardinal anastomosis, 2) intersupracardinalanastomosis VSSCA: ventral subsupracardinal anastomosis, DSSCA: dorsal subsupracardinal anastomosis, RK: right kidney, LK: left kidney, AA: abdominal aorta

The development of the LRV is quite complex, particularly compared to the development of the right renal vein. This complexity is because the LRV is closely connected to the IVC during its development, involving intricate embryonic processes. The initial fourth to eighth weeks of the embryonic period are crucial for the emergence of the IVC and LRV [1,2]. The paired longitudinal posterior cardinal vein, subcardinal, and supracardinal venous channels are involved in the origination of the IVC and LRV [1,2]. All of these veins are connected through anastomotic channels [1,2]. The subcardinal and supracardinal veins sprout from the posterior cardinal vein and located medial to it. The subcardinal vein remains anterior to the aorta, while the supracardinal vein remains posterior. The posterior cardinal veins primarily function during the first six weeks. After this period, they begin to regress, and their functions are further maintained by subsequently emerging venous channels, namely, subcardinal and supracardinal [1,2]. During the eighth week of human embryogenesis, the LRV forms a circumaortic venous ring or renal collar (Figure 7).

**Figure 8:**
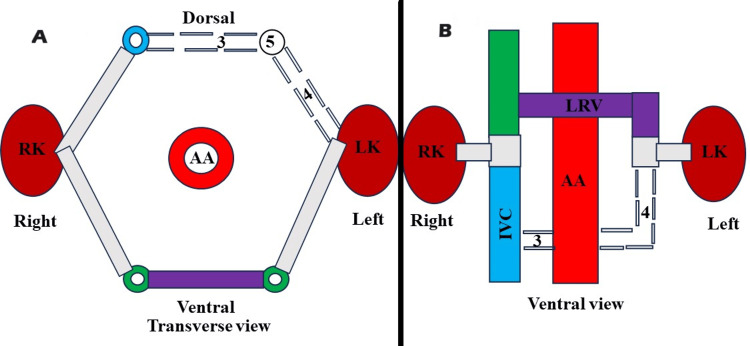
A: Schematic representation of degeneration of the retroaortic venous limb of circumaortic venous collar or renal collar (transverse view). 3: indicates the degeneration of intersupracardinal anastomosis, 4- obliteration of the left dorsal subsupracardinal anastomosis, 5- regression of the left supracardinal vein. B- Depicts a schematic representation of degeneration of the retroaortic venous limb of circumaortic venous collar or Renal collar (Ventral view). 3- indicates degeneration of intersupracardinal anastomosis, 4: obliteration of the left dorsal subsupracardinal anastomosis. Formation of the left renal vein (LRV). LK: left kidney, RK: right kidney, AA: abdominal aorta, IVC: inferior vena cava

Through the ventral intersubcardinal anastomosis, dorsal intersupracardinal anastomosis, and lateral subsupracardinal anastomosis, the ventral arch of the renal collar passes anterior to the aorta, and the dorsal arch passes posterior to the aorta (Figure 7). Later on, the dorsal arch regresses, and only the ventral arch persists, giving rise to the future normal LRV (Figure 8).

**Figure 9:**
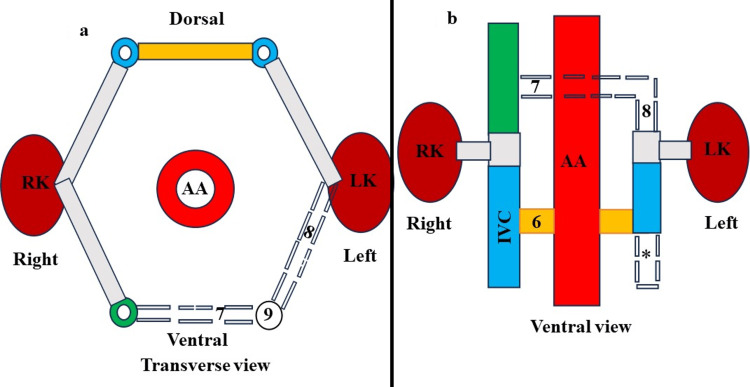
A) Schematic representation of the degeneration of the preaortic venous limb of circumaortic venous collar or renal collar (transverse view). 7: degeneration of intersubcardinal anastomosis, 8: degeneration of the left ventral subsupracardinal anastomosis, 9: regression of the left subcardinal vein. B) Schematic representation of the degeneration of the preaortic venous limb of the circumaortic venous collar or renal collar (ventral view). 6: formation of the retroaortic left renal vein. 7: degeneration of the intersubcardinal anastomosis, 8: degeneration of the left ventral subsupracardinal anastomosis. * indicates the regression of the remaining part of the left supracardinal vein. LK: left kidney, RK: right kidney, AA: abdominal aorta, IVC: inferior vena cava

The LRV is formed by the contribution of the intersubcardinal anastomosis and the left ventral subsupracardinal anastomosis, as well as the regression of the intersupracardinal anastomosis and left dorsal subsupracardinal anastomosis. The RLRV forms due to the persistence of the dorsal venous arch of the circumaortic venous collar or renal collar and regression of the ventral venous arch (Figure 9).

Finally, the RLRV (Figure 9) emerges due to the persistence of the intersupracardinal anastomosis and the left dorsal subsupracardinal anastomosis. Simultaneously, there is a regression of the intersubcardinal anastomosis and the left ventral subsupracardinal anastomosis [10,13,14,15]. The plexiform venous network of the posterior cardinal, subcardinal vein, and supracardinal vein undergoes frequent remodeling, giving rise to diverse alternative morphological and topographical characteristics of the LRV [1]. During the embryonic period, the kidney initially forms in the sacral region [1,2]. Then, between the sixth and ninth week of gestation, it moves upward to its designated lumbar position [1,2]. At the same time, significant remodeling occurs in the veins in the abdominal region. If the left kidney fails to move to its typical higher position during this period, it may lead to various venous anomalies associated with the left kidney [1]. Hence, we opine that errors in the normal relocation of the left kidney also contribute to the emergence of the protean drainage pattern of the left kidney.

The authors of earlier articles have mentioned various types of RLRV. It seems that these authors have individually studied and categorized the RLRV based on its anatomical variations. This classification system assists in distinguishing between different types of RLRV based on their connection points, and it can be valuable in medical research and surgical procedures involving the renal veins. The developmental anomalies of the LRV can be categorized into four types [3,16]: In type 1, the preaortic venous arch of the circumaortic venous collar degenerates, and the retroaortic venous arch subsists and drains into the IVC in the normal position (0.3-1.9%). In type 2, the anterior venous arch of the circumaortic venous collar degenerates. The posterior venous arch becomes RLRV and course obliquely and joins the gonadal and ascending lumbar vein before draining into the IVC at L4-L5 vertebral level (0.4-0.9%). In type 3, the CLRV has subsistence of intersubcardinal anastomosis, intersupracardinal anastomosis, and lateral subsupracardinal anastomoses of veins (1.5-8.7%). In type 4, it gives rise to a RLRV, which courses obliquely downwards and runs posterior to the distal part of the aorta to join the left common iliac vein (0.16%). It is a rare congenital anomaly. Some authors have proposed a two-type system to classify the RLRV [16,17]. In type I, the RLRV joins the IVC horizontally without additional connection. In type II, the RLRV drains into the IVC obliquely at the level of L4 to L5 after joining the gonadal and ascending lumbar veins (additional connection). Others have classified the RLRV into four main types [3,16]: In type 1, the RLRV drains transversally into the IVC. In type 2, the RLRV drains obliquely into the IVC at the L4-L5 vertebral level after joining the ascending lumbar and gonadal veins. In type 3, the CLRV is formed. The presence of both the pre- and retroaortic venous segment of the CLRV both drain into the IVC. In type 4, the RLRV has an oblique, downward trajectory and joins the left common iliac vein.

Among the different protean drainage patterns of the LRV, the CLRV is crucial during various retroperitoneal surgeries. Considering the clinical significance of the CLRV, it is further classified considering the surgical aspect. According to Gillot, there are three main types of CLRV [18]: In type 1, the CLRV emerges from the renal hilum as a single venous channel. It partially bifurcates slightly distal to the renal hilum. The retroaortic venous limb drains separately to the IVC after joining the hemiazygos vein. In type 2, the CLRV consists of two separate venous channels emerging from the renal hilum. Both venous segments unite anterior to the aorta and drain into the IVC as a single venous channel. It is a relatively common variety. In type 3, a true CLRV consists of ventral aortic and dorsal aortic venous limbs that originate separately from the renal hilum and drain independently into the IVC. It is further categorized into two subtypes: (3a) inferior polar (the dorsal aortic venous segment is located below and the ventral aortic venous segment is positioned above) and (3b) superior polar (the superior vein acquires the dorsoaortic position and a downward oblique course toward IVC). The inferior ventral aortic venous limb is transversely placed. The adrenal and gonadal veins drain into it. In Figure 1, we proposed it as a different type of CLRV. This finding was somewhat similar to the type 2 category as recommended by Gillot [18]. The remodeling happened due to the persistence of intersubcardinal anastomosis, left subsupracardinal anastomosis, and obliteration of the intersupracardinal anastomosis. In Figure 2, This congenital anomaly appeared due to the intersubcardinal anastomosis, left subsupracardinal anastomosis, left supracardinal vein, and degeneration of the intersupracardinal anastomosis. In Figure 3, the probable explanation for this rare congenital LRV topography might be the degeneration of the intersubcardinal anastomosis, intersupracardinal anastomosis, left ventral subsupracardinal anastomosis, and persistence of the left supracardinal vein and left dorsal subsupracardinal anastomosis.

The incidence of the CLRV and RLRV varies remarkably in different sets of people (cadaveric/clinical). Kuzan et al. reported the incidence of RLRV and CLRV as 1.84% and 0.7%, respectively, following abdominal CT scans of 12,341 patients [5]. Hostiuc et al. found the incidence of RLRV to be 3% and CLRV to be 3.5% after analyzing 115 articles in a meta-analysis [6]. Yi et al. conducted a meta-analysis of the literature to determine the incidence of CLRV and RLRV in cadaveric individuals, finding rates of 0.6-17.0% and 0.5-3.5%, respectively. They also found incidence rates of 0.1-10.0% for CLRV and 0.4-9.3% for RLRV in clinical populations [19]. Table 2 illustrates the incidence of RLRV, CLRV, and RILRV in cadaveric dissection [17,18,20,21,22,23].

**Table 2:** Incidence of the RLRV, CLRV, and RILRV in cadavers. RLRV: retroaortic left renal vein, CLRV: circumaortic left renal vein, RILRV: retroiliac left renal vein

References	No. of cadavers	RLRV (%)	CLRV (%)	RILRV (%)
Hoeltl et al. [17]	354	1.1	0.6	-
Gillot [18]	322	2.5	5.6	-
Okamoto [20]	70	0.7	6.3	-
Anson and Daseler [21]	100	1	1	-
Davis and Lundberg [22]	270	1.8	1.5	-
Reis and Esenther [23]	500	2.4	6	-
Present study	21	-	2 cases	1 case

A recent literature review has revealed that in more than 90% of cases of laparoscopic donor nephrectomy, the left kidney has been procured preferential to the right kidney. Reasons for this paradigm shift in practice are many. A longer LRV compared to the right side technically facilitates subsequent transplantation. The short and thin-walled right renal vein often causes difficulty in venous anastomosis during the transplantation procedure. There are increased chances of iatrogenic vascular injury and vasospasm during donor nephrectomy as the right renal artery is directly posterior to the short and thin-walled right renal vein, thus requiring increased intraoperative manipulation of the renal artery. Few studies on laparoscopic right donor nephrectomy have even mentioned higher chances for vascular complications with eventual graft loss as compared to left donor nephrectomy [12].

Clinical implications

Discussing RLRV cases, one of our patients, a 23-year-old male, presented with left-sided aching testicular pain due to varicocele, along with on-and-off left flank pain and microscopic hematuria. On CT angio evaluation, he had RLRV type II. Microscopic hematuria in these cases is caused by “posterior nutcracker syndrome” (NCS), due to constriction of RLRV between the aorta and vertebra, which in turn leads to venous hypertension resulting in rupture of the thin-walled septum separating the veins from the urinary collecting system. The left gonadal vein joins the LRV at a right angle, resulting in the drainage of the left gonadal vein under higher pressure compared to the right gonadal vein, which drains into the IVC at an acute angle and experiences comparatively lower pressure. In addition, the posterior nutcracker syndrome, where the RLRV is compressed by the abdominal aorta, leads to backflow and further dilation of the left gonadal vein, ultimately causing a varicocele. Gibo and Onitsuka described the case of a 13-year-old girl presenting with macrohematuria and low back pain; during the clinical investigation, it was found to have an RLRV, with compression of the vein between the aorta and the spine, causing an increased pressure gradient between the LRV and the IVC [8]. During such constriction, multiple venous collaterals develop with gonadal, ascending lumbar, adrenal, ureteral, and capsular veins. This vascular channel may lead to varicocele and sometimes infertility [19]. RLRV is associated with pelvic congestion syndrome in females portrayed by lower abdominal pain, dysmenorrhea, dyspareunia, emotional lability, and gluteal or thigh varices again due to posterior NCS, leading to venous hypertension and retrograde flow in ipsilateral ovarian and uterine veins [3,6,24].

In another patient with retroaortic renal vein type I with a left poorly functioning kidney, the renal vein was dissected up to the lateral border of the aorta and clipped using two hemolock clips (Weck® clips) on the patient side and one clip on the specimen side. The renal artery was clipped using hemolock clips similarly. Venous hypertension due to nutcracker syndrome in these cases may be one of the causes of renal damage. In the third patient, a 26-year-old female, RILRV type IV was the cause of PUJ obstruction leading to left flank pain, recurrent UTI, and microscopic hematuria. Heidler S et al. have also mentioned that RLRV can cause ureteropelvic junction obstruction [25]. The pelvis was intrarenal; it was dissected, dismembered, and transposed anteriorly to the vein while preserving the vein, and pyeloplasty was performed in an Anderson-Hynes manner. Both the patients were completely relieved after the procedures. There are increased chances of renal vein injury during lower pole and upper ureter dissection in these cases due to unexpected extremely low renal vein position as CT angio is rarely done for pyeloplasty. RLRV may have a high number of lumbar retroperitoneal tributaries, forming complex retroaortic systems, which can be easily injured during surgical dissection. In the presence of RLRV, the standard surgery protocol for renal transplantation and abdominal aortic aneurysm resection has to be altered especially in cases with type 2 and 4 RLRV, as they drain obliquely downward toward L5 or common iliac vein. If the surgeon is not cautious in these cases, it may get inadvertently injured during dissection of the ureter and gonadal vein near the lower pole. When operating on patients with CLRV, it has been often observed that the anterior limb of CLRV is of normal caliber, so the operating surgeon assumes it to be a single renal vein as he is unaware of the retroaortic component until and unless CT angio has been done and thoroughly studied by a radiologist and surgeon. Although we know it is rarely done in cases of simple nephrectomy and pyeloplasty, it may lead to inadvertent injury to the posterior limb, extensive hemorrhage, and sometimes death during the procedure like nephrectomy, abdominal aortic aneurysm repair, and donor nephrectomy. As far as cases with multiple renal veins are concerned, they are associated with higher chances of injury during donor nephrectomy resulting in the need for repair during bench surgery and prolonged ischemia time. They are often associated with a higher risk of thrombosis in graft renal veins. They often need bench surgery with either side-to-side anastomosis followed by anastomosis to the recipient's external iliac vein as a single common vein, thus increasing the cold ischemia time of graft. They may be anastomosed separately to the recipient's external iliac vein but at the cost of increased warm ischemia time [26].

The transperitoneal approach is the best for CLRV, and the retroperitoneoscopic approach is the best for RLRV. In cases of RLRV, the retroperitoneal working space is limited, even though left and right retroperitoneal laparoscopic donor nephrectomies can be performed safely in the hands of an expert surgeon. Retroperitoneoscopically a long left retroaortic renal vein can be easily harvested. The laparoscopic surgeon must be familiar with the retroperitoneal approach and its technical difficulty before attempting retroperitoneoscopic left donor nephrectomy in the presence of a retroaortic renal vein. Medial dissection of the renal vein should be carefully performed as it is the step during which most injuries occur but is extremely necessary for better visualization of vein course and tributaries. In cases of CLRV, the gonadal vein is first identified and traced toward the LRV where the two components of the circumaortic vein are identified and precisely dissected. The decision is wisely made regarding which component of the CLRV should be preserved for the subsequent renal transplantation. The posterior component of the CLRV usually has a smaller caliber and can be safely controlled with clips. Therefore, if we are comfortable controlling large lumbar renal vein branches laparoscopically, a similar technique is used to control a circumaortic renal vein during donor nephrectomy. The adrenal vein, gonadal vein, and lumbar vein are identified, clipped, and divided. The renal artery is then dissected circumferentially and prepared for subsequent clipping [27]. Most authors have reported that there was no clinically significant difference regarding parameters such as operative time, warm ischemia time, and estimated blood loss in patients with circumaortic or retroaortic renal veins when compared to normal renal vein anatomy [7]. Overzealous dissection of renal vein anomalies may lead to a higher incidence of postoperative pancreatitis and inadvertent injury to the superior mesenteric artery or celiac axis. During aortic aneurysm surgery control of the proximal aorta may be difficult to achieve in patients with a circumaortic venous ring, especially posterior component is injured at its junction with the IVC during dissection. Injury to the retroaortic segment has been reported to be as high as 40%. Hemorrhage has led to transection and retraction of the proximal aorta; to prevent this, temporary division of the anterior portion of the circumaortic ring has been suggested by some authors. Precise medial dissection and skeletonization of renal veins are required for an accurate visualization of tributaries, such as adrenal, lumbar, and gonadal veins, and thus proper identification and control of retroaortic or circumaortic components [28].

## Conclusions

Understanding the variable anatomical drainage pattern of the LRV is crucial for diagnosing and treating chronic symptoms and preventing complications during surgery. The presence of a circumaortic or retroaortic renal vein does not represent a contraindication to simple or live donor nephrectomy. Even in the presence of an anomalous LRV anatomy, a renal allograft can be procured and transplanted with excellent donor and recipient outcomes. Preoperative detection of an LRV anomaly can decrease morbidity and mortality associated with injury to this variant. However, the imaging and its interpretation may not always be flawless, and these anomalies can be missed so the surgeon should be cautious and ready for such unforeseen venous anomalies and ready to manage them successfully without any surgical mishap.
